# Behavioral ecology: New technology enables a more holistic view of complex animal behavior

**DOI:** 10.1371/journal.pbio.3002264

**Published:** 2023-08-24

**Authors:** Gail L. Patricelli

**Affiliations:** Department of Evolution and Ecology, University of California, Davis, California, United States of America

## Abstract

Animal behavior is notoriously complex. This Perspective looks at how technological advances have enabled the study of variability over space and time, enabling a more holistic view of this complexity and expanding the focus from single components to behavioral systems.

## Teaser

As any animal observer will tell you, behavior is complex. A more holistic view of this complexity is emerging as technological advances enable the study of spatiotemporal variability and expand the focus from single components to behavioral systems.

This article is part of the *PLOS Biology* 20th Anniversary Collection.

Behavior is more than just a suite of traits; it is the crux where the inside of the organism meets and interacts with the external environment. On the inside of the organism, behavior emerges through an interaction of genetic, physiological, cognitive, and developmental processes, which can be affected, in turn, by that organism’s behavior and experience. Behavior is also how organisms respond to—and influence—the biotic and physical environment, which includes potential mates, rivals, offspring, group members, predators, prey, and pathogens—all with their own behaviors—interacting amidst changing seasons and climates. And behaviors may manifest at multiple scales, from individuals to swarms. For the past 20 years and going forward, many of the exciting frontiers in the study of animal behavior involve grappling with this complexity in a more holistic way, examining the causes and functions of variability in behavior over space and time, and scaling up from components to systems, examining interaction networks that function as a whole.

Many breakthroughs on key aspects of animal behavior (social behavior, mate choice, communication, predator–prey dynamics, foraging ecology, and migration) have been enabled by advances in technology that allow us to collect detailed and simultaneous data from many components of complex systems (Box 1). These tools are made possible by increased computing power, rapid advances in machine learning, and the development of smaller, cheaper, and more powerful hardware. Such tools are pushing the study of behavior, like other fields of science, into the “big data” era.

**Box 1. Technology allowing a more holistic view of animal behavior.**

Advances in both hardware for collecting data and machine learning software to analyze those data are expanding the detail and the scale at which we can study behavior.

**Hardware**

- Animal-borne telemetry tags, which collect or transmit data about movements and other measures, can be miniaturized to much less than a gram and provide precise locations with onboard GPS or data from sensors (e.g., accelerometers, physiological monitors, microphones, or light-level monitors). Tags may store or transmit data to other tags, land-based receiver arrays, or satellites (e.g., ICARUS or MOTUS). These tags reveal aspects of animal lives that were previously unobservable, helping to identify critical resources and habitats for protection (e.g., migration corridors and refueling sites), exposure and response to stressors (e.g., human activity, noise and light pollution), and cryptic behaviors (e.g., nocturnal movements, quiet communication, and visits to potential mates).
- Other key hardware includes synchronized microphone arrays to triangulate animal positions from the arrival time of their vocalizations [1], drones with imaging tools, terrestrial laser scanning (ground-based LiDAR) for detailed habitat measures, and Passive Integrated Transponders (PIT tags).

**Machine learning**

- Supervised machine learning, trained on human-annotated data sets, is automating tedious tasks and making detailed analysis of large datasets more feasible.
- Unsupervised machine learning can identify new patterns in movement tracks and other behavioral data, providing insights less limited by human biases and reducing (not eliminating) subjective decisions about which characteristics to measure.
- On videos, freely available software [2] uses machine learning to track position and orientation on multiple individuals, enabling the study of social networks and swarm dynamics. Machine learning can also be used for pose estimation by tracking the relative position of multiple body parts for biomechanical studies of behaviors (e.g., DEEPLabCut [3]).
- On audio recordings, machine learning is automating detection and identification of sounds from birds, bats, and other vocal animals, enabling acoustic monitoring over time, in remote locations, and at night [4] and increasing the feasibility of using synchronized microphone arrays to study vocal behavior and movements [1].

Spatiotemporal variability is a ubiquitous feature of animal behavior. By necessity, behaviors are often measured by choosing a few key characteristics that can be scored accurately and repeatably, often averaging multiple measures from consistent conditions. This allows behaviorists to examine, for example, the relationship between courtship rate and mating success, or dominance hierarchies in social groups. While important, there is increasing awareness that fascinating biology is being averaged away, such as differences among individuals in the ability to execute behaviors consistently or adapt to changing social and environmental situations, or variation among groups in the stability of social networks [5–7]. The past few decades have seen frameworks for understanding aspects of this behavioral variation, such as consistent individual differences (CIDs) and personality, behavioral reaction norms, and dynamic social network analyses, but the difficulty of collecting data has limited the scope of empirical work.

To capture and analyze variability itself, we need enough snapshots to make a movie, multiple measures of behaviors within and among individuals or groups, across time and context, so the patterns of change can be examined. New hardware and machine learning algorithms for tracking movements and recognizing patterns are opening exciting new opportunities for collecting such data [2,4,8].

For example, using GPS telemetry tags in the wild or overhead video in captive enclosures, it is increasingly feasible to study the causes and consequences of CIDs in behavior, such as activity level or aggressiveness, by tracking multiple individuals throughout development or among contexts. Patterns of behavioral variation can then be examined relative to genotype, epigenetics, experience, adult behavior (of the focal animal, their parents, and their offspring), and social group dynamics. In fish, for example, tracking has revealed that CIDs in behavior among clonal mollies raised in identical conditions are present from birth and strengthen over time [9] and that CIDs among sticklebacks in sociability and boldness can affect the movement and foraging performance of entire shoals [10].

Similar machine learning algorithms can track the position of body parts for pose estimation, automating frame-by frame analysis of biomechanics during courting, fighting, prey capture, locomotion, and other behaviors [3,8]. This can save time, expand the number of traits measured on focal or interacting individuals, and reduce subjectivity in analyses (Box 1). These opportunities for high-resolution data collection will (I hope) inspire further development of theory in neglected areas, such as optimal tactics during courtship and other dynamic behavioral interactions [5].

New tracking tools are also helping us to scale up from spatiotemporal analyses of behavioral components to a systems-level view of the whole. The systems approach focuses on structure–function relationships, moving from cause-and-effect thinking to synergistic thinking, by emphasizing interactions, linkages, and integrated phenotypes [5,11].

For example, a hot topic of research for more than 20 years has been why sexual selection frequently favors complex courtship displays with components in different sensory modalities, combining songs, dances, colors, scents, and vibrations [5,6,11]. A recent comparative analysis of the famously complex and spectacular displays of 40 species of birds-of-paradise utilized video and audio recordings, as well as color patterns from museum skins, finding positive relationships instead of trade-offs between complexity in the acoustic, color, and behavioral display components [12]. The authors argued that integrated suites of traits evolve as a courtship phenotype, with functional overlap and interdependency providing robustness and promoting diversification. Further research is needed to determine whether similar patterns emerge in the complex courtship displays of other clades of birds, as well as clades of reptiles, amphibians, fishes, insects, and spiders. With machine learning tools for automated data collection, such broad comparative analyses are becoming possible with growing online databases, such as libraries of audio and video recordings and 3D scans of museum specimens. Ultimately, to understand the evolution of complex courtship phenotypes, as in birds-of-paradise, we must also understand how male display components interact to stimulate the females’ sensory, cognitive, and motivational systems to influence their mate choice. In other words, a holistic approach is also required to understand the aesthetic experiences and complex preferences of the females these courtship displays evolved to impress. This interface between behavioral ecology and neuroethology promises exciting discoveries about the evolution of some of nature’s most beautiful spectacles.

Systems-level analyses of multicomponent social groups have been similarly insightful. Tracking of large groups of birds is revealing surprisingly complex, multilevel social systems, from families, to cohesive groups of unrelated individuals, to fission–fusion dynamics among groups, to structured flocks of interacting species [13]. Tracking is also allowing the detailed examination of collective behaviors [10,14], exploring how behavioral rules followed by individuals scale into emergent properties of groups, such as swarming behavior of locusts. For example, by modelling how group size and spacing affect individuals’ views through the crowd, researchers are learning how geometry affects swarm dynamics and collective decisions.

Along with benefits of new technology, come challenges. To name a few, minimizing the impacts of our technology on animal bearers, finding meaningful biology in the output of black box algorithms, and not letting data volume and high statistical power substitute for thoughtful experimental design and biologically relevant effect sizes. Downloading data from satellites is no substitute for time in the field or lab learning about natural history and carefully observing behaviors, which is essential to inspire creativity and anchor us to the real world. At its best, new technology complements existing methods and helps to reveal hidden dimensions of behavior. Moving into the big data era, animal behavior, like other fields, can minimize pitfalls by increasing transparency, standardization, and sharing of data, algorithms, and statistical code.

As the pace of urbanization, habitat loss, climate change, and other human impacts increase, behavior will often be the first response, either allowing animals to adjust to change, or not. Behavioral changes are often the first signs scientists can measure as evidence of human impacts. Behavior is also what often inspires public fascination and concern about wildlife. Therefore, in addition to addressing basic questions about behavioral evolution, new technology and a more holistic view of animal behavior is key to understanding, predicting, and mitigating human impacts on wildlife. For example, behaviorists are revealing how noise and light pollution impact social behaviors, improving methods for population monitoring and restoration, and reducing human–wildlife conflict. The next 20 years will bring increased opportunity and increased necessity for animal behaviorists to engage actively with conservationists, policy makers, stakeholders, and the public to find solutions to these complex problems.
